# Research Progress on Regulatory Mechanisms of Conical Cell Morphogenesis in *Arabidopsis thaliana*

**DOI:** 10.3390/plants15132069

**Published:** 2026-07-03

**Authors:** Xinfei Li, Deshu Lin, Lilan Zhu

**Affiliations:** Basic Forestry and Proteomic Research Center, Haixia Institute of Science and Technology, Fujian Agriculture and Forestry University, Fuzhou 350002, China; xinfei@fafu.edu.cn

**Keywords:** *Arabidopsis thaliana*, petal conical cells, cortical microtubules, auxin, cell wall acidification, cell morphogenesis

## Abstract

Flowering plants are universally adorned with conical epidermal cells on their petals, which play a pivotal role in their function. They modulate the petal microenvironment by regulating wetting and temperature homeostasis and enhance pollinator attraction through tactile signaling. Despite their ecological and physiological significance, the molecular mechanisms that regulate the development of their distinct conical shape remain largely unknown. This review synthesizes recent advances in *Arabidopsis thaliana* (*A. thaliana*) research to elucidate the regulatory network governing conical cell morphogenesis. We summarize the recently established live confocal imaging approach for investigating conical cell morphogenesis and the core regulatory pathways elucidated thus far: the spatiotemporal orchestration of the cortical microtubule arrays, governed by the PP2A-KATANIN and ANGUSTIFOLIA-ROS modules. Furthermore, auxin-mediated cell wall acidification also plays a critical role in conical cell morphogenesis. Building upon these established regulatory modules, integrating computational modeling and uncovering new regulatory components in future research will profoundly enhance the value of conical cells as a system for studying plant cell morphogenesis. This will enable researchers to decipher the intricate biochemical signaling mechanisms that act in concert to orchestrate plant cell morphogenesis.

## 1. Introduction

Plant epidermis, originating from the protodermis of meristematic tissues, forms the vital outermost barrier of aerial organs, such as stems, leaves, and flowers, and underground structures like roots, acting as a critical interface between plants and their surrounding environment [1,2,3]. Composed of densely packed cells boasting specialized structures, gas-exchanging stomata, hair-like trichomes, elongated hypocotyl cells, intricately interlocking jigsaw-puzzle pavement cells, and conical-shaped epidermal cells, epidermal tissue fulfills multiple vital roles. Diverse plant epidermal cells safeguard the plant from water loss, mediate essential gas exchange [4], withstand mechanical stresses [5], defend against pathogen invasion [6], and facilitate the perception of environmental signals [7]. Among the diversity of specialized epidermal cells, conical cells on petals stand out as an iconic evolutionary identity unique to flowering plants (angiosperms) [8,9,10], especially prevalent in animal-pollinated species. These cells are distinguished by their remarkable conical-to-pyramidal morphology, broad-based with convex apices, and certain species are further embellished with exquisitely intricate, nanoscale ridge-like ornamentations across their surfaces [10,11], which amplifies their structural complexity.

The functional significance of petal conical cells extends beyond structural uniqueness, integrating ecological and physiological roles that are critical for plant reproductive success and environmental adaptation [11,12]. Their conical morphology increases the roughness of the petal surface, providing essential tactile cues and a stable grip for pollinators like bees [13]. This, in turn, reduces slipping and guides efficient foraging. Additionally, the three-dimensional structure of conical cells sculpts light reflection, intensifying petal color contrast and their visual allure to pollinators [14]. Meanwhile, certain species amplify aroma-related enzymes within these cells to heighten scent emission [15]. Importantly, the dense arrangement of conical cells, acting synergistically with the cuticle layer, may effectively curtail water loss and bolster defenses against both biotic and abiotic stresses. This crucial mechanism preserves the structural integrity and functional capacity of petals throughout the flowering phase. Given that the production of a variety of horticultural crops relies upon biotic pollination, the traits of conical cells exert a direct influence on pollination efficiency and crop yield, highlighting their considerable practical significance.

Cell morphogenesis, the fascinating process by which cells acquire their intricate and specific shapes, stands as a fundamental enigma in plant developmental biology. This process is orchestrated mainly by the dynamic interplay of cytoskeletal organization, precise cell wall modification, intricate hormonal signaling, and mechanical forces [16,17,18,19,20,21]. Among these factors, the microtubule cytoskeleton plays a pivotal role in determining anisotropic cell expansion. As dynamic polymers, cortical microtubules guide the deposition of cellulose microfibrils within the cell wall, thereby dictating the directionality of cell expansion and ultimately regulating anisotropic cell expansion [22,23,24,25,26]. For instance, during the morphogenesis of *A. thaliana* pavement cells, microtubule arrays undergo spatiotemporal reorganization in response to developmental and mechanical cues to drive cellular interdigitation [19,27,28,29,30]. Another fundamental mechanism governing plant cell morphogenesis is the classic “acid growth theory”. This theory proposes that auxin signaling initiates apoplast acidification by activating plasma membrane (PM)-localized H^+^-ATPases. The resulting decrease in apoplastic pH then activates cell-wall-loosening enzymes, which work in concert with turgor pressure to drive cellular expansion [31,32,33,34,35]. Initially introduced five decades ago, this powerful concept has garnered substantial experimental support across diverse cell types, including hypocotyl cells and cotton fibers [36,37,38]. In these systems, maintaining an optimal apoplastic pH proves indispensable for sustained cell expansion.

Despite the ecological and physiological significance of conical cells, deciphering their molecular regulatory network has long remained elusive. This challenge was overcome only with the establishment of *A. thaliana* conical cells as an experimental model [39,40]. Serving as a model plant with extensively annotated genetics and straightforward experimental manipulation, particularly live imaging, *A. thaliana* has facilitated the dissection of core pathways governing conical cell morphogenesis [39,40]. In recent years, live confocal imaging, integrated with genetic and biochemical experiments, has demonstrated that protein phosphatase 2A (PP2A) directly interacts with KATANIN, a conserved microtubule-severing protein [41]. This PP2A-KATANIN module mediates microtubule organization, playing a crucial role in sculpting the sharpened tip and establishing the final conical cell shape [39,42]. Moreover, ANGUSTIFOLIA (AN), a key regulator of plant cell morphology [43,44], orchestrates reactive oxygen species (ROS) homeostasis to govern microtubule organization. This AN-dependent pathway further engages in genetic crosstalk with the KATANIN-mediated microtubule pathway [45]. Furthermore, auxin-dependent cell wall acidification serves as a critical pathway regulating conical cell morphogenesis [46]. Despite these advances, our understanding is still incomplete regarding the precise molecular mechanisms that regulate microtubule organization and mediate auxin signaling to promote proper conical cell morphogenesis. Moreover, it is unclear whether these pathways are conserved across diverse plant species. Building upon these foundational advances and enduring questions, this review synthesizes recent breakthroughs in *A. thaliana* research to describe the regulatory network guiding conical cell morphogenesis while also probing the persistent challenges and unresolved mysteries that lie ahead. Furthermore, we propose that both biochemical and biomechanical signaling act as critical regulators in conical cell morphogenesis, and we highlight crucial future research directions integrating computational modeling.

The scientific significance of this work lies in two key aspects: First, it positions *A. thaliana* conical cells as a good system for investigating fundamental principles of plant cell morphogenesis, effectively supplementing established cellular systems like root hairs, pavement cells, and trichomes. Second, it provides insight into translational research by optimizing conical cell traits to enhance pollination efficiency and stress resistance and ultimately offers novel strategies for crop breeding and improvement.

## 2. Developing a Live Confocal Imaging Method to Investigate Conical Cell Morphology

The study of petal conical cell morphogenesis has long been constrained by technical limitations in visualizing dynamic cellular processes. For decades, scanning electron microscopy (SEM) remained the gold standard for analyzing conical cell morphology, largely relying on fixed, dehydrated samples to capture high-resolution surface structures [47,48]. This imaging approach yielded foundational discoveries: early work in *Antirrhinum majus* (*snapdragon*) identified the R2R3-MYB transcription factor MIXTA as a master regulator of conical cell development [12,47]. Loss-of-function mutations in *MIXTA* resulted in flattened petal epidermal cells, reduced tactile guidance for pollinators, impaired foraging efficiency, and a dramatic decline in seed set [11,12,13,47]. Subsequent studies in *Arabidopsis thaliana*, *Petunia × hybrida*, and *Phalaenopsis* validated the evolutionary conservation of MYB family proteins in orchestrating conical cell-shape formation [10,48,49,50,51], reinforcing the centrality of this transcriptional module. However, SEM’s inherent limitations—an inability to capture subcellular dynamics and a lack of temporal resolution—severely restricted mechanistic investigations. Unlike model systems for plant cell morphogenesis, such as trichomes, root hairs, and pavement cells, where live-cell imaging via confocal laser scanning microscopy (CLSM) integrates cell biology and genetics, conical cell research remains largely detached from confocal imaging analysis of fundamental processes like cytoskeletal organization or signaling dynamics. This technical gap impeded a deeper understanding of whether and how regulators integrate upstream developmental signals to translate genetic blueprints into downstream targets, and how precise spatiotemporal regulation of cytoskeletal organization is achieved.

**Figure 1:**
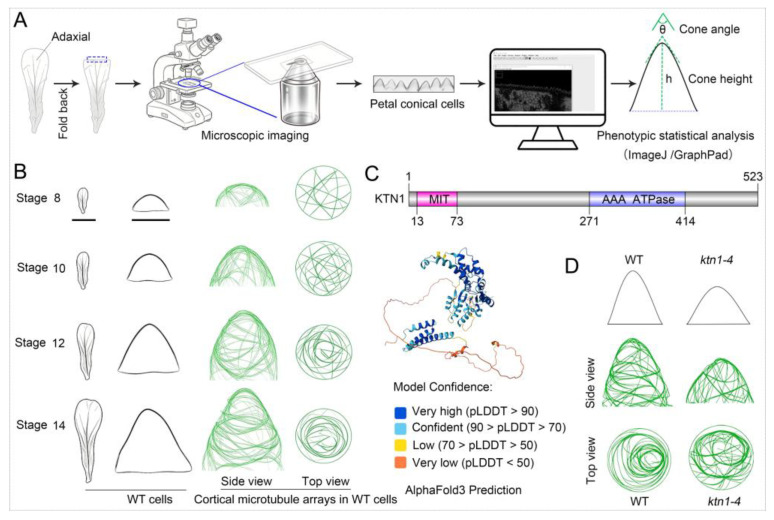
Schematic illustration of conical cell morphogenesis and microtubule reorganization in *A. thaliana* petals. (**A**) A schematic diagram depicts the side view and distinct morphological features of conical cells from a folded *A. thaliana* petal. The apical cone angle and height of these cells were precisely measured and quantified using ImageJ software, version 2016. (**B**) Schematic cartoons illustrate petal conical cells and microtubule organization at key developmental stages of the wild type. Both side and top views depict the microtubule arrays within these wild-type conical cells from stages 8, 10, 12, and 14 petals. The scale bar in the left panel is 1 mm, while in the right panel it is 10 µm. (**C**) A schematic diagram depicts the KTN1 protein and its structure as predicted by AlphaFold 3. (**D**) Schematic cartoons illustrate the striking contrast in microtubule arrangements at key developmental stages between wild-type and *ktn1-4* mutant conical cells.

Since 2017, a method based on a confocal microsopy or fluorescence microscopy, suitable for imaging the side view of *A. thaliana* petal conical cells, was pioneered [39,40,52]. The crucial breakthrough emerged with the folded-petal mounting technique (Figure 1A): carefully folding freshly dissected petals, taken from developing flower buds at stages 8–14, as defined by a previous study [39,40], along their midlines. This technique enabled the lateral imaging of conical cells using either confocal or fluorescence microscopy (Figure 1A). This innovation addressed two critical limitations: while conventional flat mounting captured only the apical, top-down perspective, precluding measurements of cell height and apex angle, the folding method simultaneously preserved cell viability and stabilized the tissue. This allowed for extended time-lapse imaging without compromising cellular integrity. Combined with quantitative image analysis pipelines (e.g., ImageJ/Fiji plugins, version 2016), this method enabled precise and dynamic characterization of conical cell morphometrics, including the apical angle (θ) and base-to-apex height (h) across successive developmental stages (Figure 1A).

## 3. KATANIN-Mediated Microtubule Reorganization—Shifting from Disordered Arrays into Distinct Helical Bundles—Is Required for Conical Cell Morphogenesis

A pivotal breakthrough in employing the live confocal approach to study conical cell morphogenesis was the integration of fluorescent cytoskeletal markers within this imaging assay [39]. By expressing microtubule-specific markers, such as GFP-α-tubulin6 (GFP-TUA6), alongside actin filament markers like GFP-fABD2 in conical cells, the subcellular distribution of the cortical microtubule and actin cytoskeleton was examined separately during conical cell morphogenesis [39]. This combination of live-cell imaging and genetic labeling enables the mechanistic dissection of the cytoskeletal contributions to conical cell-shape determination. Confocal imaging revealed a developmentally regulated transition in cortical microtubule organization, directly correlating with conical cell morphogenesis. During early flower developmental stages, conical cells from stage 8 remain undifferentiated, exhibiting a flattened, plate-like morphology; concurrently, cortical microtubules display a disordered arrangement across the cell surface [39] (Figure 1B). As petals progress into stages 10 and 11, marking the onset of conical anisotropic cell expansion, when observed from the top of the cells, microtubules undergo a dramatic reorganization: they shift from disordered arrays into distinct helical bundles that spiral tightly around the cell’s apical–basal axis (Figure 1B). This ordered helical arrangement persists through stage 14, coinciding with the rapid elongation and tapering of the cell apex, ultimately generating the characteristic conical shape (Figure 1B). Genetic and pharmacological manipulations further validated microtubules as a critical driver of conical cell-shape formation. Long-term treatment of developing flower buds (stages 7–8) with the microtubule-depolymerizing agent oryzalin resulted in the complete abolition of pyramid-like conical cell formation upon reaching stage 14. Instead, the cells developed into swollen, spherical forms entirely lacking apical tapering [39].

In plants and animals, KATANIN is assembled from two subunits, p60 and p80 [53,54,55]. The p60 subunit features an N-terminal microtubule-interacting and trafficking domain (p60-MIT), connected via a linker to a C-terminal ATPase domain that houses the enzyme’s catalytic activity [56]. X-ray structures of the AAA ATPase of KATANIN in monomeric nucleotide-free and pseudo-oligomeric ADP-bound states elucidated conformational changes within the AAA subdomains, revealing the structural basis for the KATANIN heterododecamer’s instability [57]. The swift dissociation of AAA oligomers may trigger an autoinhibited state, thereby preventing inappropriate microtubule-severing activity. Alternatively, the cyclical disassembly of these complexes into heterodimers may itself contribute to the microtubule-severing mechanism. In *A. thaliana*, p60 KATANIN (KTN1) has been shown to mediate microtubule severing, specifically at sites of microtubule nucleation and crossover [41,58,59]. Using AlphaFold 3 sever [60], the p60 KTN1 structure was predicted (Figure 1C). We suggest that p60/p80 KTN1 form a heterodimer–heterotetramer that may diffuse along microtubule lattices, bind ATP, and sever microtubules, which needs to be further investigated in future studies [56,57]. Notably, loss-of-function mutations in *A. thaliana KTN1* effectively prevented microtubule reorganization during conical cell morphogenesis [39]. The *ktn1* mutants retained disorganized microtubule arrays throughout later developmental stages, leading to conical cells with blunt, expanded apices and increased apical angles [39] (Figure 1D). These findings support a model in which KTN1-mediated microtubule severing and reorganization are essential for establishing the helical arrays that guide anisotropic cell expansion—restricting lateral growth while promoting apical elongation—during conical cell morphogenesis [39] (Figure 1D). However, the subcellular localization of KTN1 and its association with microtubules, specifically how KTN1-dependent microtubule-severing events generate the well-ordered helical array during conical cell morphogenesis, remain to be elucidated.

In contrast to cortical microtubules, the role of actin filaments (microfilaments) in conical cell morphogenesis remains ambiguous. Live imaging of actin revealed disordered cortical actin networks [39]. However, pharmacological disruption of actin or loss-of-function mutations in the ARP2/3 actin nucleation factors failed to yield distinct conical cell phenotypes; these cells retained a normal height and apex angle [39]. This indicates that actin might not act as a primary driver of the conical cell shape, or its function could be redundant with alternative pathways. Future studies utilizing higher-resolution imaging or conditional actin mutants may resolve this ambiguity by revealing subtle defects in vesicular trafficking or cell wall deposition that remain invisible to conventional CLSM.

In summary, the establishment of live confocal imaging has fundamentally reshaped our understanding of conical cell morphogenesis, transforming it from a static, descriptive discipline into a vibrant, mechanistic field. By enabling the visualization of the cytoskeleton, this approach has established microtubule reorganization into well-ordered arrays as the central driver of conical cell-shape formation.

## 4. AN Regulates Conical Cell Morphogenesis by Coordinating ROS Homeostasis and Microtubule Organization

The establishment of live confocal imaging has not only enabled the dynamic observation of conical cell morphogenesis but also facilitated genetic screens to identify regulators of conical cell morphogenesis. Through a genetic screen, AN, a plant-specific protein homologous to animal C-terminal binding protein/Brefeldin A-ADP ribosylated substrate (CTBP/BARS) [43,44], emerged as a critical modulator of the conical cell shape [45]. AN was primarily characterized for its roles in leaf and trichome development. Loss-of-function *an* mutants exhibit narrow leaves, reduced trichome branching, and simplified pavement cell morphology, phenotypes distinctly linked to enhanced cortical microtubule alignment [43,44]. Intriguingly, *an* mutants exhibit petal conical cells with significantly enlarged apical angles and blunted apices [45]. This phenotype is closely associated with disorganized cortical microtubule arrays, presenting a striking contrast to the enhanced microtubule alignment typically observed within the pavement cells of cotyledons or leaves [43,44,61]. This cell-type-specific regulation highlights the functional versatility of AN and underscores the uniqueness of the conical cell morphogenesis program.

**Figure 2:**
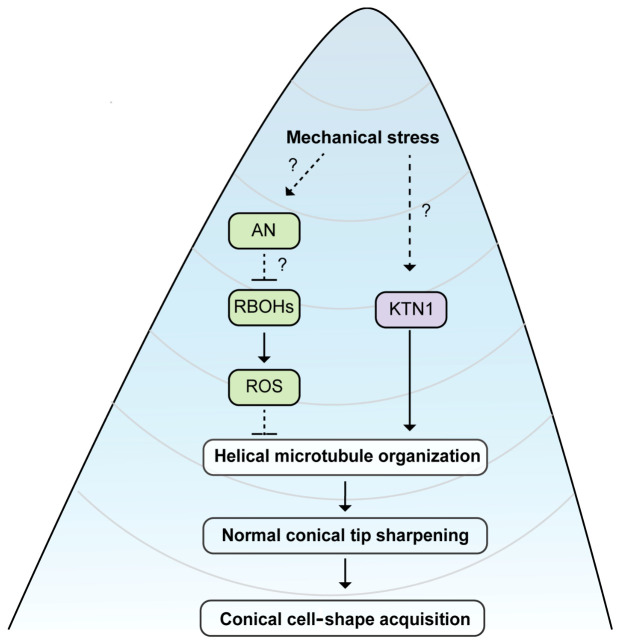
The AN–ROS module acts synergistically with KTN1 to regulate microtubule arrangement and conical cell morphogenesis. This schematic model depicts the regulatory mechanism by which the AN–ROS module controls microtubule patterning and tip sharpening of conical cells. AN functions in concert with KTN1 to modulate microtubule organization and conical cell-shape acquisition. We propose that AN directly modulates RBOH activity to suppress ROS accumulation, a mechanism that warrants further investigation. Perturbed ROS homeostasis hinders the formation of orderly circumferential microtubule arrays. Mechanical stress is hypothesized to trigger the activity of both AN and KTN1. Pale ring-shaped filaments inside the cell denote cortical microtubules. Question marks represent unknown regulatory links, dashed lines stand for unclarified pathways, and solid arrows indicate confirmed positive regulatory effects. Double-sided dashed lines denote putative uncertain negative regulation.

Given that the loss of *AN* function triggers elevated ROS accumulation in leaves via an unknown mechanism [62], we propose that similarly aberrant ROS buildup in *an* mutant petals may be responsible for their wider-angled conical cell tips. ROS, a crucial regulator of plant cell shape, functions by modulating cytoskeletal organization and cell wall modification [63,64]. By employing ROS-specific fluorescent probes, the levels of superoxide anion (O_2_^−^) and hydrogen peroxide (H_2_O_2_) in *an* mutant conical cells were assessed [45]. Dihydroethidium (DHE) staining for O_2_^−^ and 2′,7′-dichlorodihydrofluorescein diacetate (H_2_DCF-DA) staining for H_2_O_2_ demonstrated a marked increase in both O_2_^−^ and H_2_O_2_ within the *an* mutants compared with the wild type [45]. This finding links AN to ROS homeostasis, suggesting that AN functions to restrain ROS accumulation in conical cell morphogenesis. Exogenous application of H_2_O_2_ to wild-type flower buds (stages 7–8) disrupted the developmental transition of microtubules from disordered arrays into helical alignments within conical cells. As these buds progressed to stage 14, the disruption resulted in conical cells with enlarged apical angles and flattened apices, effectively mimicking the *an* mutant phenotype [45]. These data reveal a causal chain: the loss of *AN* function triggers ROS overaccumulation, which leads to microtubule disorganization, ultimately culminating in impaired conical cell apex formation [45] (Figure 2).

To further explore the regulatory network downstream of AN, an ethyl methanesulfonate (EMS) mutagenesis screen in the *an* mutant background was performed to identify enhancers of the conical cell phenotype [45]. This screen resulted in the identification of *KTN1*, previously demonstrated to be essential for promoting helical microtubule formation in conical cells [39], as a genetic interactor of *AN*. The *an ktn1* double mutants exhibited a synergistic enhancement of the conical cell-shape defect [45]. Compared to single mutants, double-mutant cells were drastically swollen, with a complete loss of conical shape. Confocal imaging revealed that microtubule arrays in the double mutant were severely disorganized, lacking any semblance of helical alignment [45]. This indicates that *AN* and *KTN1* act in parallel or partially overlapping pathways to promote microtubule ordering. These genetic data support a unified model for conical cell morphogenesis: the AN–ROS–microtubule module and the KTN1–microtubule organization pathway function cooperatively to establish the helical microtubule arrays required for anisotropic cell expansion (Figure 2). KTN1 mediates microtubule severing and reorganization, while AN restricts ROS accumulation to prevent ROS-induced microtubule disruption. Together, these pathways orchestrate the precise spatiotemporal control of microtubule organization, steering the transition from flattened to conical morphology. This synergy underscores the intricate complexity of cytoskeletal regulation within conical cells, where multiple modules collaboratively converge to fine-tune microtubule behavior.

Despite these advances, questions still remain regarding the AN’s molecular function and its integration into conical cell morphogenesis. How AN modulates ROS homeostasis remains elusive. Given AN’s localization to stress granules and the PM [65,66], and considering that prior studies have identified ROS-producing enzymes, specifically the PM-localized NADPH oxidases, known as respiratory burst oxidase homologs (RBOHs), which serve as major sources of ROS in plant cells [67,68], as candidate interacting proteins of AN-GFP via a liquid chromatography–tandem mass spectrometry (LC-MS/MS) assay (as detailed in the supplemental data [45]), we hypothesize that AN may directly interact with RBOHs (Figure 2). Future studies should examine whether AN directly regulates the activity of RBOHs through physical interaction, or alternatively modulates ROS scavenging pathways, such as catalases and peroxidases, to sustain ROS homeostasis.

While the AN–ROS module has been demonstrated to regulate microtubule organization during petal conical cell shaping [45], its specific influence on the cell wall throughout this process remains largely unexplored. Emerging evidence from other expanding plant cells suggests that apoplastic ROS may modulate cell wall mechanics [69,70]. On the one hand, hydroxyl radicals can promote cell wall loosening through the nonenzymatic cleavage of wall polysaccharides, such as pectins and xyloglucans, thereby enhancing wall extensibility and facilitating cell expansion [71,72,73,74,75]. On the other hand, H_2_O_2_ can be utilized by peroxidases to drive the oxidative cross-linking of phenolic compounds, extensins, and other structural wall components, leading to wall stiffening and consequent growth restriction. Therefore, the spatial and temporal balance of ROS production and scavenging is likely pivotal for maintaining an optimal level of wall plasticity during conical cell morphogenesis. In petal conical cells, we propose that ROS influence cell shape through at least two interconnected pathways. First, ROS homeostasis may modulate cell wall architecture indirectly by orchestrating cortical microtubule organization, which, in turn, guides the deposition of cellulose microfibrils and directs anisotropic cell expansion. Second, apoplastic ROS may directly regulate cell wall extensibility by influencing the remodeling of wall polysaccharides and the activity of key cell wall-modifying proteins, such as expansins, xyloglucan endotransglycosylases/hydrolases, pectin methylesterases, and peroxidases. Specifically, alterations in pectin methylesterification and peroxidase-mediated cross-linking can modify wall porosity, stiffness, and local yielding capacity, thereby directly shaping the transition from early isotropic expansion to later tip sharpening. However, direct biochemical evidence linking ROS levels to cell wall composition and wall-loosening enzyme activity within petal conical cells remains unknown. Future studies that integrate live ROS imaging, cell wall immunolabeling, pectin methylesterification analysis, and cell-type-specific transcriptomic or proteomic approaches will be crucial for elucidating how ROS-dependent wall remodeling coordinates with microtubule-based morphogenesis.

Intriguingly, AN exhibits completely opposite regulatory effects on cortical microtubule organization in two typical plant epidermal cell types: it negatively modulates microtubule alignment in leaf pavement cells but promotes ordered microtubule arrangement in petal conical cells, and this functional divergence can be largely attributed to cell-type-specific regulatory contexts and distinct downstream effector partnerships of AN. In cotyledon pavement cells, AN functions through a defined protein cascade, where it physically interacts with IPGA1, and IPGA1 further associates with the microtubule-severing protein KTN1 to jointly disrupt ordered microtubule alignment, thereby facilitating the interdigitated growth of pavement cells [61]. In contrast, the working mechanism of AN in petal conical cells relies on a distinct ROS-dependent regulatory pathway rather than the IPGA1-KTN1 module. In conical cells, AN acts as a critical negative regulator of ROS overaccumulation, maintaining intracellular ROS homeostasis to stabilize cortical microtubule arrangement and support the polarized, upright morphogenesis of conical cells [45]. Notably, unlike the AN–IPGA1–KTN1 regulatory axis in pavement cells, direct interactions between AN and microtubule-associated downstream effectors remain unclarified during conical cell development. The absence of the IPGA1-mediated microtubule regulatory cascade, combined with the unique ROS-based regulatory microenvironment in petal epidermal cells, fundamentally reshapes AN’s regulatory role in microtubule patterning, resulting in its cell-type-specific divergent functions in plant cell morphogenesis. Given that KTN1-mediated microtubule reorganization plays a role in the mechanical stress-driven alignment of microtubules [27], we propose that mechanical stress may concurrently activate both KTN1 and AN protein functions (Figure 2), a hypothesis that warrants further exploration in future studies.

In summary, AN emerges as a central coordinator of conical cell morphogenesis, integrating both ROS homeostasis and microtubule organization with KTN1-mediated cytoskeletal regulation (Figure 2).

## 5. The PP2A-KTN1 Module Promotes Microtubule Ordering and Conical Cell Morphogenesis

The identification of KTN1 as a key regulator of microtubule reorganization in conical cells raised critical questions about how its cellular abundance is spatiotemporally regulated during conical cell morphogenesis. Post-translational modifications (PTMs), particularly phosphorylation and dephosphorylation, have been demonstrated to control the function, stability, and subcellular localization of certain microtubule-associated proteins (MAPs) [76,77]. Notably, in the nematode *Caenorhabditis elegans*, protein phosphorylation and dephosphorylation are crucial for regulating the activity and protein stability of KATANIN [78,79,80]. However, the key protein kinases or phosphatases mediating KTN1 phosphorylation remained undiscovered. Using a Phos-tag sodium dodecyl sulfate (SDS)-polyacrylamide gel mobility shift assay, both phosphorylated and dephosphorylated forms of KTN1 were identified in *A. thaliana* [42]. This widely employed technique effectively separates distinct phosphorylated and non-phosphorylated derivatives of a protein [81]. Total proteins were extracted from a transgenic line expressing *pKTN1::KTN1-6 × His-4 × Myc* using Phos-tag gel electrophoresis and were detected by immunoblot analysis with an anti-Myc antibody. Two distinct bands were observed, with the upper, slow-migrating band designated as the phosphorylated form (P+), and the lower band as the non-phosphorylated form (P−) of the protein [42]. The phosphorylated form of KTN1 shifted predominantly to the position of the non-phosphorylated form in a λPPase dosage-dependent manner. Furthermore, PhosSTOP (a proprietary blend of phosphatase inhibitors) effectively inhibited λPPase-mediated KTN1 dephosphorylation [42]. Importantly, a recent study has revealed that N-terminal phosphorylation of the *A. thaliana* KTN1 acts as a critical regulatory switch that controls microtubule severing during both vegetative and reproductive development [82]. Through the use of *in vitro* biochemical assays, phosphorylation was found to occur concurrently at three conserved serine residues (specifically S92, S147, and S199) [82]. However, the identity of the kinases that directly phosphorylate KTN1 at these three serine residues remains unknown. Candidate protein kinases, such as Aurora kinases or cyclin-dependent kinases, which may act as KTN1’s kinases in plants, are yet to be explored [82]. Together, these results demonstrate that KTN1 undergoes cycles of phosphorylation and dephosphorylation in *A. thaliana*.

To elucidate the regulatory network governing KTN1 function, we employed a GFP-Trap-immunoprecipitation strategy coupled with LC-MS/MS [42], using a KTN1-GFP fusion protein as the molecular bait. This was followed by rigorous tandem affinity purification using leaves harvested from 4-week-old *p35S::KTN1-GFP* transgenic plants. This screen identified subunits of the PP2A complex as prominent KTN1-interacting partners. PP2A represents a highly conserved serine/threonine protein phosphatase, functioning as a core heterotrimeric complex in eukaryotes [83,84]. This essential complex comprises three distinct core subunits: a structural scaffolding A subunit (PP2AA), a regulatory B subunit (PP2AB), and a catalytic C subunit (PP2AC) [83]. In *A. thaliana*, the PP2A family is significantly expanded, featuring multiple isoforms of each subunit; for instance, three A subunits, seventeen B subunits, and five C subunits [85]. This diversity allows for the formation of numerous distinct holoenzymes, each possessing unique substrate specificities and critical developmental functions. PP2A complexes govern a broad spectrum of essential plant processes, encompassing hormonal signaling, cytoskeletal dynamics, cell cycle progression, and stress responses [86,87,88,89,90,91,92,93]. Crucially, however, prior to this study, no direct link between PP2A and KTN1 had been established in plants, and the role of PP2A in conical cell morphogenesis remained entirely unexplored.

To confirm the PP2A-KTN1 interaction identified by IP-MS, in vitro and in vivo protein–protein interaction assays were performed. Yeast two-hybrid (Y2H) analysis revealed a direct physical interaction between the regulatory B subunit TONNEAU2 (TON2, a B″-type PP2AB subunit) and KTN1 [42], consistent with the formation of a functional PP2A holoenzyme that binds KTN1. Furthermore, co-immunoprecipitation (Co-IP) assays in both human HEK293T cells and *A. thaliana* revealed the in vivo interaction between KTN1 and either RCN1, PP2AA2, PP2AA3, TON2, PP2AC3, or PP2AC4, indicating KTN1’s association with the PP2A holoenzyme [42]. In vitro dephosphorylation assays utilizing purified recombinant PP2A holoenzyme and immunoprecipitated KTN1-GFP revealed that PP2A directly dephosphorylates KTN1. This activity was clearly evidenced by a decreased P+/P− ratio following incubation of the PP2A complex, purified from an *A. thaliana* transgenic line expressing GFP-RCN1, with KTN1-GFP [42]. Strikingly, cantharidin markedly inhibited the PP2A complex’s dephosphorylation of KTN1-GFP. To explore whether PP2A regulates KTN1 dephosphorylation, transgenic lines expressing *pKTN1::KTN1-6 × His-4 × Myc* were crossed with *pp2a* double mutants, specifically *rcn1 pp2aa2*, *rcn1 pp2aa3*, and *rcn1 pp2aa3*, and the resulting lines were generated for conducting Phos-tag analysis. The P+/P− ratio was significantly higher in the *pp2a* double mutants compared to the wild type, indicating elevated KTN1 phosphorylation levels in these mutants [42]. These results established that PP2A directly regulates KTN1 phosphorylation in *A. thaliana*.

To elucidate the functional consequences of PP2A-mediated KTN1 dephosphorylation, KTN1 protein stability was subsequently analyzed in *PP2A*-deficient mutants [42]. Western blot analysis showed that KTN1 protein levels were significantly reduced in *pp2aa1 pp2aa2* double mutants (deficient in two A subunits) and a *TON2 RNAi* line compared to wild type [42]. This demonstrates that PP2A-mediated dephosphorylation enhances KTN1 protein stability, likely by blocking phosphorylation-dependent proteasomal degradation, which is consistent with previous reports indicating phosphorylation of the *C. elegans* microtubule-severing protein KATANIN often targets it for ubiquitination and subsequent degradation [78,79,80]. Genetic analysis of the *pp2a* mutants further confirmed its role in conical cell morphogenesis. Both *pp2aa1 pp2aa2* double mutants and *TON2 RNAi* lines exhibited severe conical cell defects, markedly more pronounced than those observed in *ktn1* mutants: conical cells were drastically stunted, featuring widened apical angles and a complete loss of pyramidal tapering [42]. Confocal imaging of microtubule markers in these mutants revealed profoundly disorganized cortical microtubule arrays, completely devoid of helical alignment throughout development [42]. This mirrors the impaired microtubule reorganization observed in *ktn1* mutants, yet is markedly more severe. These data establish a pathway: PP2A dephosphorylates and stabilizes KTN1, facilitating KTN1-mediated microtubule severing and helical array formation, which ultimately drives anisotropic conical cell expansion. Notably, the more pronounced phenotype of *pp2a* double mutants compared to *ktn1* single mutants suggests that PP2A likely targets additional substrates beyond KTN1 that contribute to conical cell morphogenesis. For example, PP2A dephosphorylates other MAPs, which could cooperate with KTN1 to fine-tune microtubule dynamics. Future studies identifying PP2A substrates within conical cells, for instance, via phosphoproteomic comparison of wild type and *pp2a* mutants, will crucially help delineate the full scope of PP2A’s regulatory network. The discovery of the PP2A-KTN1 module marks a significant advance in plant cell morphogenesis: it represents the first report of KTN1 regulation via dephosphorylation in plants.

**Figure 3:**
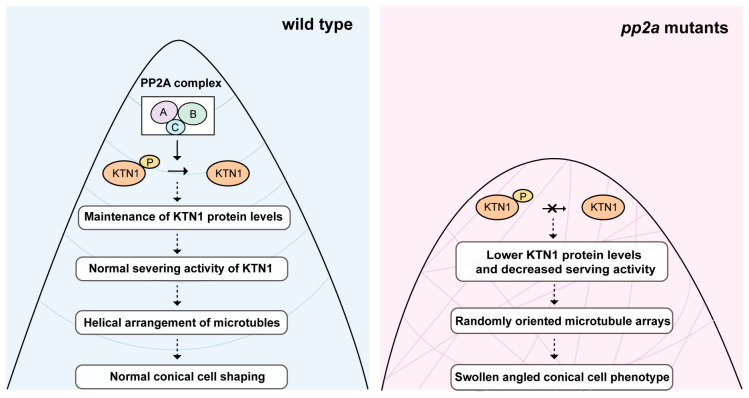
The PP2A-KTN1 regulatory module controls cortical microtubule patterning and conical cell morphogenesis. Schematic model depicting PP2A-dependent modulation of KTN1 function and downstream cellular morphogenesis. In wild-type conical cells (**left panel**), PP2A directly catalyzes KTN1 dephosphorylation to dynamically adjust its phosphorylation state throughout developmental progression. Such PP2A-mediated post-translational modification preserves KTN1 protein abundance, presumably by inhibiting its ubiquitin-proteasomal degradation [42]. The functional PP2A-KTN1 complex facilitates orderly assembly and circumferential arrangement of cortical microtubules, steering directional cellulose deposition and supporting intact conical cell shaping. In *pp2a* loss-of-function mutants (**right panel**), impaired KTN1 dephosphorylation disrupts protein stability, further diminishing the microtubule-severing capacity of KTN1. Disorganized microtubule architecture ultimately triggers aberrant cell expansion and swollen conical cell morphology. Pale filaments inside the cell denote cortical microtubules. Dashed arrows represent putative uncertain positive regulation, while solid arrows indicate confirmed positive regulatory effects.

Based on these findings, a working model was proposed that delineates the PP2A-KTN1 module-driven processes of microtubule organization and conical cell morphogenesis (Figure 3). In this model, PP2A physically interacts with KTN1 to form the PP2A-KTN1 module, which catalyzes the dephosphorylation of KTN1 by PP2A. This PP2A activity functions in maintaining KTN1 protein stability by preventing its targeted proteasomal degradation (Figure 3). Consequently, this stabilization may empower the proper severing activity of KTN1, promoting microtubule reorganization into circumferential cortical arrays and thereby driving the conical anisotropic expansion essential for normal conical cell morphogenesis (Figure 3). Despite these advances, several pressing questions remain unanswered. First, the specific phosphorylation sites within KTN1, targeted by PP2A, remain unidentified. Mapping these sites will require mass spectrometric analysis of KTN1 phosphopeptides; subsequently, site-directed mutagenesis, converting phosphorylatable serine/threonine residues to non-phosphorylatable alanine or phosphomimetic aspartate, will clarify their critical role in regulating KTN1 stability and microtubule-severing activity. Second, the kinase(s) responsible for initiating KTN1 phosphorylation are still unknown. Third, the precise molecular mechanism by which PP2A-mediated dephosphorylation stabilizes KTN1, potentially by inhibiting interactions with E3 ubiquitin ligases, demands further exploration.

In summary, the PP2A-KTN1 module represents a central regulatory pathway governing microtubule organization and conical cell morphogenesis. By revealing the critical role of dephosphorylation in KTN1 stability and function, this work significantly advances our understanding of the post-translational mechanisms that precisely fine-tune cytoskeletal dynamics during plant cell-shape determination. Crucially, resolving these unanswered questions will not only deepen our knowledge of conical cell morphogenesis but also provide broader insights into the roles of PP2A-KTN1 signaling throughout eukaryotic development.

## 6. AUXIN Mediates Apoplastic pH Modification to Regulate Conical Cell Morphogenesis

While microtubule reorganization into helical aligned arrays constitutes a core regulatory axis for later stages during conical cell morphogenesis [39], the mechanism of earlier conical anisotropic cell expansion, likely driven by cell wall loosening and turgor pressure, remains incompletely addressed. The acid growth theory, proposed over five decades ago, posits that auxin-triggered apoplastic acidification activates cell wall-loosening enzymes (e.g., expansins, xyloglucan endotransglucosylase/hydrolases [XTHs]) and likely weakens cellulose–pectin interactions, thereby promoting cell expansion or elongation [31,32,33,34,35]. This theory has been validated across an array of cell types, including *A. thaliana* root hairs, hypocotyls, and cotton fibers [36,37,38], yet its relevance to conical cell morphogenesis, particularly during the critical early stages of shape formation, remained unexplored until 2020. To explore whether acid growth promotes conical cell-shape formation, quantitative apoplastic pH imaging combined with genetic and pharmacological manipulations of auxin signaling was performed [46]. This study uncovered a pathway wherein auxin-mediated apoplastic acidification orchestrates both the outgrowth and tapering processes in conical cells [46]. A critical technical prerequisite for investigating acid growth in conical cells was the ability to measure apoplastic pH with spatial and temporal resolution. 8-hydroxypyrene-1,3,6-trisulfonic acid trisodium salt (HPTS), a ratiometric, pH-sensitive fluorescent dye [37], was used to quantify pH changes during conical cell morphogenesis [46]. HPTS exhibits pH-dependent excitation spectra (peak shifts from 405 nm to 458 nm as the pH decreases), enabling ratiometric imaging (458 nm/405 nm emission) to generate quantitative pH maps in both root and hypocotyle cells via confocal microscopy [37]. Employing the folded-petal mounting technique for HPTS staining, the apoplastic pH within conical cells was captured and measured across flower developmental stages 8–14 [46]. This approach revealed a developmentally regulated apoplastic acidification event, tightly synchronized with the onset of conical cell morphogenesis. At stage 8 (undifferentiated flattened cells), the apoplastic pH averaged 5.5 ± 0.04; strikingly; by stage 9 (initiation of conical cell outgrowth perpendicular to the petal surface), the pH had decreased to 5.1 ± 0.02 [46]. Subsequently, throughout stages 9 and 11 (active tapering), the apoplastic pH gradually rebounded, reaching 5.4 ± 0.02 in mature conical cells at stage 11. Strikingly, beyond stage 11, the cells had already attained a conical morphology; concurrently, the apoplastic pH remained unaltered [46]. Notably, although conical anisotropic cell expansion persists beyond stage 11, the cone angles of the cells remained largely unchanged [39]. Previous research demonstrated that microtubules reorient from random to well-ordered circumferential arrays after stage 11 [39], a process instrumental in modulating conical cell tip sharpening. This finding may partially elucidate why conical cell expansion after stage 11 requires well-ordered microtubules to reinforce the cell wall but not cell wall acidification.

To investigate whether apoplastic pH patterns regulate the conical cell shape, developing petals were treated with pharmacological agents, specifically Fusicoccin (FCC) or N,N′-dicyclohexylcarbodiimide (DCCD) [46]. These treatments alter H^+^-ATPase activity, thereby generating lower and higher apoplastic pH values, respectively. FCC activates plasma membrane-localized H^+^-ATPases [94], whereas DCCD inhibits the activity of these H^+^-ATPases [95]. Long-term FCC treatments significantly reduced apoplastic pH while enhancing tip sharpening and increasing cone heights of conical cells [46]. Consistent with this observation, conical cells of the *OPEN STOMATA2* (*ost2-2*) mutant, which expresses the constitutively active form of the H^+^-ATPase AHA1 [96], displayed a significantly reduced apoplastic pH. These cells also exhibited a distinct conical cell phenotype, characterized by notably narrower tip angles and substantially greater cone heights compared to the wild type [46]. In contrast, DCCD treatment prompted a rise in pH and produced a distinct phenotype characterized by dramatically wider tip angles and significantly reduced cone heights within conical cells compared to mock-treated petals [46]. These findings strongly suggest that conical anisotropic cell expansion is primarily driven by apoplastic acidification.

It has been demonstrated that auxin promotes apoplastic acidification by activating H^+^-ATPases in roots and hypocotyls [97]. To determine whether auxin signaling governs pH modification in conical cells, the auxin response reporter R2D2 (Ratiometric version of 2 D2s) was used. This transgenic line expresses a combination of RPS5A-driven Venus-tagged auxin degradable reporter protein (DII:n3 × Venus) and an RFP-tagged undegradable protein (mDII:ntdTomato), enabling the quantitative analysis of auxin responses [98]. The nuclear auxin signaling accumulation is captured through the fluorescence ratio of the mDII/DII signal, where an elevated ratio signifies increased auxin activity. Confocal imaging of R2D2-expressing petals revealed a sharp increase in nuclear auxin signaling from stage 8 to stage 9, strikingly coinciding with the onset of apoplastic acidification and conical cell outgrowth [46]. This temporal correlation suggests that auxin signaling precedes and likely triggers apoplastic acidification. Notably, the mDII/DII ratio decreased significantly from stage 9 to 11 during active tip sharpening, returning to stage 8 levels by stage 11, and remained stable thereafter as the conical cells underwent radial and longitudinal expansion [39,46]. These data demonstrate a tight spatiotemporal correlation between auxin signaling dynamics and the initial anisotropic expansion of conical cells.

To genetically validate the requirement of auxin signaling for apoplastic acidification and conical cell morphogenesis, multiple auxin receptor mutants were analyzed [46]. Auxin binding to TIR1/AFB nuclear receptors triggers the ubiquitination and proteasomal degradation of AUX/IAA repressors, releasing ARF transcription factors to activate auxin-responsive gene expression [99,100]. Strikingly, both triple and quadruple auxin receptor mutants exhibited significantly elevated apoplastic pHs and markedly reduced cone heights, yet maintained normal cone angles compared to wild-type plants. Pharmacological inhibition of TIR1/AFB receptors using auxinole also increased apoplastic pH and induced defective conical cell morphogenesis with wider tips and shorter cones [46]. Similarly, chemical inhibition of auxin biosynthesis using yucasin [5-(4-chlorophenyl)-4H-1,2,4-triazole-3-thiol], a specific inhibitor of YUCCA flavin monooxygenases [101], or perturbation of polar auxin transport with NPA (naphthylphthalamic acid), resulted in apoplastic alkalization and impaired conical cell expansion. These results demonstrate that auxin biosynthesis, polar auxin transport, and TIR1/AFB-dependent auxin signaling are all essential for apoplastic acidification and normal conical cell morphogenesis.

Since TIR1/AFB signaling operates through ARF transcription factors [99], it is plausible that members of the ARF family regulate the development of conical cells. Among the 23 *A. thaliana* ARFs, ARF6 and ARF8 are known master regulators of flower development, as the *arf6-2 arf8-3* double mutant exhibits severe floral defects, including arrested petal growth [102]. Previous work reported a complete loss of conical cell outgrowth in *arf6-2 arf8-3* petals [103], but the underlying mechanism remained unknown. Phenotypic and pH analyses confirmed that *arf6-2 arf8-3* epidermal cells remain completely flattened and display a significantly elevated apoplastic pH [46]. These results identify the ARF6/ARF8 module as an essential downstream component of auxin signaling that mediates the transition from cell division to differentiation and initiates conical cell morphogenesis via apoplastic acidification.

Auxin exerts a well-documented biphasic, concentration-dependent effect on cell growth, promoting expansion at low concentrations and inhibiting growth at high concentrations in a tissue-specific manner [104,105,106]. To test whether auxin levels directly modulate apoplastic pH and conical cell expansion, stage 8 floral buds were treated with exogenous IAA (100 and 200 μM). Long-term IAA application significantly reduced apoplastic pH and induced enhanced tip sharpening and increased cone height [46]. Consistently, the *35S::iaaM* transgenic line, which exhibits elevated endogenous auxin biosynthesis [107], displayed reduced apoplastic pH, narrower cone angles, and taller cones compared to the wild type [46]. To establish the epistatic relationship between auxin and apoplastic acidification, rescue experiments were performed using the H^+^-ATPase inhibitor DCCD. Co-treatment of wild-type petals with IAA and DCCD abolished auxin-induced tip sharpening, instead causing isotropic swelling of conical cells [46]. Similarly, DCCD treatment of *35S::iaaM* plants suppressed the enhanced conical cell phenotype and induced cellular swelling [46]. These genetic and pharmacological epistasis assays establish that auxin, at least in part, governs the anisotropic expansion of conical cells by inducing H^+^-ATPase-dependent apoplastic acidification.

**Figure 4:**
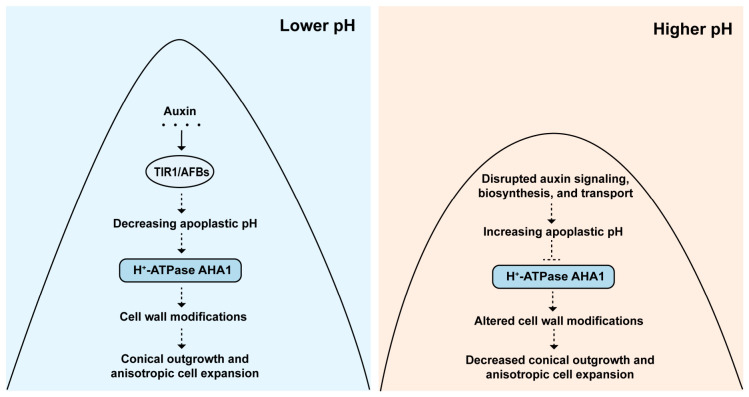
Auxin modulates apoplastic pH homeostasis to fine-tune conical cell morphogenesis. Schematic model showing auxin-governed apoplastic pH dynamics and its regulatory role in conical cell development. On the left panel, auxin perception by TIR1/AFB receptor complexes triggers downstream signaling cascades that activate plasma membrane-localized H^+^-ATPase AHA1. Functional AHA1 drives cell wall acidification and facilitates cell wall structural remodeling, which collectively facilitates apical protrusion and maintains proper anisotropic expansion of conical cells. In contrast, the right panel depicts perturbed auxin metabolism, signal transduction, or polar transport, which elevates apoplastic pH and compromises AHA1 proton-pumping activity. Impaired cell wall remodeling consequently restricts conical tip elongation and distorts directional cell expansion. Dashed arrows represent putative uncertain positive regulation, while solid arrows indicate confirmed positive regulatory effects. Double-sided dashed lines denote putative uncertain negative regulation.

Building on these findings, a working model was proposed [46], in which auxin signaling, likely via the TIR1/AFBs receptor, drives apoplastic acidification to promote conical cell morphogenesis (Figure 4). The discovery that auxin-mediated apoplastic pH modification controls conical cell morphogenesis represents a major advance in understanding plant cell-shape regulation. First, this work expands the classic acid growth theory to a morphologically complex cell type, demonstrating that auxin-driven apoplastic acidification is not limited to elongated cell types such as hypocotyls and root hairs, but also contributes to the formation of specialized three-dimensional cell shapes. Second, these findings highlight the versatility and cell-type specificity of auxin action. While auxin universally controls cell expansion, it employs distinct downstream programs in different cell types, illustrating how a conserved signaling pathway generates diverse cellular morphologies. Despite this progress, important questions remain, including the specific H^+^-ATPase isoforms involved, the kinases responsible for their activation, and how auxin signaling interacts with cell wall integrity pathways. Future studies should aim to examine whether the cell-surface receptor kinases, TMKs, known to modulate cell wall acidification and drive cell elongation in hypocotyls [97], also play a pivotal role in regulating H^+^-ATPase activities during conical cell morphogenesis.

In conclusion, auxin-mediated apoplastic acidification acts as a pivotal early trigger for conical cell outgrowth during morphogenesis, operating in concert with the PP2A-KTN1 microtubule pathway to direct anisotropic cell expansion. Nevertheless, the precise mechanistic details await further exploration in future studies.

## 7. Discussion and Future Perspectives

Petal conical cells, a defining hallmark of angiosperm flower evolution, have risen as a model system for unraveling the molecular and cellular mechanisms governing plant cell shape determination. In the model organism *A. thaliana*, the petal presents as a deceptively simple organ, composed of distinct epidermal cell types. The adaxial epidermis is adorned with striking conical cells, while the abaxial side features relatively flat and interdigitated cells characterized by several lobes [108]. Recent studies have solidified the crucial role of microtubule organization in governing anisotropic petal shape formation. In *A. thaliana*, key genes, including *SPIKE1*, *ROP GTPases*, and *IPGA1*, have been shown to orchestrate microtubule dynamics and anisotropic cell expansion within the abaxial epidermis during petal morphogenesis [108,109,110]. By integrating live-cell imaging, genetic, and biochemical analyses, this review synthesizes pivotal recent advances in understanding the regulatory network governing conical cell morphogenesis in *A. thaliana*. First, the folded-petal live-confocal imaging assay [39,40] has overcome long-standing limitations of static SEM-based analysis, enabling visualization of subcellular processes and transforming conical cell research into a mechanistic, tractable field. Second, microtubule organization, orchestrated by the PP2A-KTN1 module and exquisitely modulated by the AN-ROS pathway, serves as the fundamental cornerstone for establishing the intricately helical, well-ordered microtubule arrays that precisely guide conical anisotropic expansion [42,45]. Third, auxin-mediated apoplastic acidification serves as a critical early signal that triggers conical cell outgrowth and tapering [46]; this validates the acid growth theory within this specialized cell type and establishes a potential link between hormonal signaling and cell wall loosening. Collectively, these findings reveal a complex yet exquisitely regulatory mechanism in which microtubule reorganization, post-translational modifications, and auxin signaling converge to sculpt *A. thaliana* conical cells. The masterful spatiotemporal division of labor among these pathways—with auxin-pH driving initial cellular outgrowth, and microtubule networks refining the final shape—ensures robust plant cell morphogenesis, while synergistic interactions (e.g., AN-KTN1, PP2A-KTN1) orchestrate essential regulatory buffering. This integrated network not only deciphers the formation of the distinct conical morphology but also illustrates the adaptive nature of plant signaling pathways in sculpting specialized cell shapes.

Despite remarkable progress, several critical frontiers still need to be explored in future studies. Among these factors, mechanical stress stands out prominently as a pivotal signaling mechanism for cell morphogenesis [18], playing an essential role in advancing our understanding of conical cell development. Plant cell morphogenesis is fundamentally regulated by the intricate interplay between biochemical signals and mechanical forces. Key physical factors, including cell wall rigidity, turgor pressure, and intercellular adhesion, collectively orchestrate precise growth dynamics [18,19,20]. While established biochemical pathways such as auxin-pH signaling and PP2A-KTN1-mediated microtubule organization operate within conical cells, the crucial role of mechanical stress signaling remains unknown. Conical cell expansion entails a precisely localized apical protrusion, a process that likely generates distinct mechanical cues, such as tensile stress within the cell wall. These mechanical signals potentially feed back to actively regulate downstream biochemical pathways. For example, mechanical stress has been shown to promote the reorganization of cortical microtubules, generating exquisitely ordered arrays within pavement cells, a process fundamentally dependent on KTN1 activity [27]. Similarly, in conical cells, intense apical expansion likely induces stress-dependent helical microtubule alignment, synergizing with the PP2A-KTN1 module to reinforce robust anisotropic cell expansion. Notably, a previous study has demonstrated that rhamnose-containing cell wall polymers modulate petal growth patterning and conical anisotropic cell expansion in a microtubule-independent manner [111], revealing a critical cell wall regulatory pathway for conical cell-shape determination. Furthermore, mechanical stress potentially activates key mechanosensitive ion channels (e.g., MSLs, OSCA1) [112,113], modulating critical apoplastic pH levels or ROS production to elegantly link physical cues directly to auxin signaling pathways or targeted cell wall loosening. Future studies must strategically combine atomic force microscopy (AFM) to precisely quantify cell wall mechanical properties with targeted genetic manipulation of mechanical signaling components, aiming to elucidate the precise mechanisms by which forces are perceived and integrated with intricate biochemical pathways throughout conical cell morphogenesis.

The remarkable complexity of conical cell morphogenesis, spanning spatiotemporal biochemical signaling, cytoskeletal organization, and likely mechanical signaling, necessitates computational modeling to integrate fragmented experimental insights into robust predictive frameworks. Finite element modeling (FEM) has proven powerful in simulating plant cell growth by incorporating cell wall mechanics and turgor pressure [114,115]. Applying three-dimensional FEM to simulate conical cell morphogenesis could help test hypotheses about the mechanical basis of apical tapering. For example, modeling the effects of microtubule-guided cellulose deposition (helical vs. random) on cell wall anisotropy, or simulating how auxin-mediated acidification alters cell wall viscoelasticity to drive apical expansion. Additionally, systems biology approaches, integrating transcriptomic, metabolomic, and cell biological data into gene regulatory networks, could identify novel regulators and crosstalk nodes between pathways (e.g., AN-ROS, PP2A-KTN1). Beyond the proposed hypothetical mechanical signaling and modeling, several molecular gaps demand further exploration: these include identifying the kinases responsible for phosphorylating KTN1 and elucidating the molecular mechanisms through which AN maintains ROS homeostasis. Future integration of experimental data with computational models will not only validate existing mechanisms but also generate testable predictions, accelerating the discovery of new regulatory principles.

Beyond its fundamental importance for understanding plant cell morphogenesis, the evolutionary conservation of the conical cell regulatory network across angiosperms, especially in crop species and pollinator-dependent plants, merits exploration to determine whether the pathways identified in *A. thaliana* are universally applicable. It is plausible that core regulators, including the KTN1-mediated microtubule reorganization pathway, the AN-ROS homeostasis module, and the auxin-dependent acid growth machinery, are broadly conserved across angiosperms. This conservation provides universal and tunable genetic targets for modifying the conical cell shape in diverse crop species. As specialized petal epidermal cells, conical cells play a pivotal role in shaping floral micro-morphology, defining petal texture, optimizing light absorption efficiency, and enhancing pollinator attraction [10,11,12,13,14]. In insect-pollinated crops, like oilseed rape and fruit trees, fine-tuning the shape, size, and distribution of these cells can refine floral architecture to maximize pollination efficiency, ultimately boosting fruit set and final yield. Moreover, the cell wall remodeling and ROS-mediated morphogenetic mechanisms uncovered in conical cells pave the way for enhancing plant organ mechanical properties and stress resilience. For ornamental flowering crops, the precise modulation of conical cell development unlocks possibilities for novel flower shapes and textures, thereby greatly enriching ornamental germplasm resources.

Mounting evidence indicates that conical cell development is closely linked to the activity of MIXTA/MIXTA-like R2R3-MYB transcription factors [51]. Functional analyses in many species, including *Antirrhinum*, *Phalaenopsis*, *A. thaliana*, and tomato, demonstrate that this regulatory module can drive anisotropic epidermal cell expansion and surface specialization [47,48,49,50,51,116,117]. *A. thaliana* conical cells offer a streamlined model for pinpointing the core regulators of epidermal cell shape. Meanwhile, comparative studies across angiosperms could illuminate how deeply conserved developmental programs have been creatively modified to produce the stunning diversity of species-specific floral surface traits. Moreover, engineering MIXTA/MYB expression to fortify cuticle integrity and refine conical cell structure could enhance a flower’s pollinator attraction while simultaneously boosting its resilience to abiotic stress. In future crop breeding, the conical cell regulatory network can serve as a valuable microscale module for precise floral design, offering novel strategies for germplasm innovation, high-yield breeding, and adaptive trait improvement in economic crops.

The study of conical cell morphogenesis reaches beyond the understanding of a single cell type—it provides a model for unraveling the fundamental principles governing plant cell-shape determination. Furthermore, their direct link to plant reproductive success and pollinator interactions positions conical cell research at the interface of developmental biology and ecology, addressing questions about how developmental traits evolve to mediate species interactions. In conclusion, the regulatory network governing conical cell morphogenesis stands as a striking testament to the intricate beauty of plant developmental systems. Future research integrating biochemical and mechanical signaling with computational modeling will revolutionize our understanding of this specialized cell type, while simultaneously advancing the fundamental principles of plant cell biology. Translational research in this field presents exciting possibilities: fine-tuning conical cell traits, such as their height or surface nanoridges, could boost pollinator attraction, fortify drought tolerance through cuticle modifications, or elevate crop yields in species that rely on pollinators.

## Data Availability

The original contributions outlined in this study are detailed within the article; for any further inquiries, please direct them to the corresponding authors.

## Abbreviations

ANGUSTIFOLIA, AN; C-terminal binding protein/Brefeldin A-ADP ribosylated substrate, CTBP/BARS; Atomic Force Microscopy, AFM; Fusicoccin, FCC; 2′,7′-dichlorodihydrofluorescein diacetate, H_2_DCF-DA; Dihydroethidium, DHE; GFP-α-tubulin6, GFP-TUA6; hydrogen peroxide, H_2_O_2_; KATANIN, KTN1; Microtubule-Associated Proteins, MAPs; N,N′-dicyclohexylcarbodiimide, DCCD; plasma membrane, PM; Protein Phosphatase 2A, PP2A; Ratiometric version of 2 D2s, R2D2; Respiratory Burst Oxidase Homologs, RBOHs; Reactive Oxygen Species, ROS; superoxide anion, O_2_^−^; Scanning Electron Microscopy, SEM; Transport Inhibitor Response 1/Auxin Signaling F-Box proteins, TIR1/AFBs; TONNEAU2, TON2; Transmembrane Receptor-like Kinases, TMKs.
